# Impact of Socioeconomic Determinants and Lifestyle Factors on the Severity of Periodontal Diseases in Adults

**DOI:** 10.7759/cureus.90716

**Published:** 2025-08-22

**Authors:** Khushboo Goel, Mukesh S Paudel, Sirjana Dahal

**Affiliations:** 1 Periodontics, Institute of Medicine, Tribhuvan University, Kathmandu, NPL; 2 Gastroenterology, National Academy of Medical Sciences, Kathmandu, NPL; 3 Community Dentistry, Institute of Medicine, Tribhuvan University, Kathmandu, NPL

**Keywords:** community obesity, diabetes type 2, periodontitis, smoking tobacco, socioeconomic factors

## Abstract

Introduction: The association between periodontal diseases with socioeconomic and lifestyle factors is multifaceted. The disparities are also well recognized. The present study aims to assess the impact of socioeconomic determinants, oral hygiene practices, and lifestyle factors on the severity of probing depths and bleeding on probing in Nepalese adults.

Methods: A total of 374 adults were included in the analysis. Independent associations of socio-demographic, oral hygiene, and lifestyle factors on dependent variables of bleeding on probing and severe periodontitis defined as ≥6 mm probing depth were determined using the chi-squared test/Fisher's exact test with multivariate and ordinal logistic regression analysis.

Results: The mean age of participants was 42.40±12.51 years. The occurrence of periodontal disease was found to be 62.3%. Multivariate analysis revealed that poor oral hygiene had a markedly increased likelihood of bleeding on probing. Bivariate analysis of probing depths revealed age, bleeding on probing, and oral hygiene status, along with lifestyle factors such as smoking and smokeless tobacco, to be statistically significant with severe probing depths. There was also an elevated risk of severe probing depths among individuals of lower socioeconomic status and their determinants. The results, however, were not statistically significant. Ordinal regression analysis revealed age with an estimate of 1.286 (p=0.001), bleeding on probing with an estimate of 0.811 (p=0.030), and additionally lifestyle factors such as current smokers with an estimate of 0.991 (p=0.002) to have a risk of severe probing depths among reported individuals.

Conclusion: Poor oral hygiene and unfavorable lifestyle factors such as smoking were strongly associated with bleeding on probing and severe probing depths. This indicates that while deep pockets are prevalent, their association with lower socioeconomic status and other lifestyle factors did not show significant changes.

## Introduction

Periodontitis, typically occurring after gingivitis, is characterized by the degradation of alveolar bone, resulting in the formation of a periodontal pocket, increased tooth mobility, and, ultimately, tooth loss [1]. As of 2021, it is estimated that approximately 1.1 billion cases were afflicted by severe periodontitis [2]. Nascimento et al. in 2024 [3] projected a significant escalation in this burden by the year 2050, with the affected population exceeding 1.5 billion including a substantial rise in both incidence and prevalence in western sub-Saharan Africa and South Asian region recording its highest rates, reaching up to 17.57% in developing countries compared to developed ones [3,4]. It is proposed that the trajectory of periodontal disease progression is influenced by various demographic factors, including age, gender, oral hygiene practices, socioeconomic status (SES) (including education, occupation, and income), as well as lifestyle determinants such as obesity, diabetes, and smoking [5].

SES is extensively correlated with oral health, with individuals from lower social strata frequently exhibiting a higher prevalence and severity of periodontal disease [6,7]. An inverse relationship has been established between the severity of periodontal disease and occupational class, with higher educational attainment and income levels being well-documented correlates [8]. These disparities may be attributable to enhanced awareness, variations in oral hygiene practices, healthier lifestyle choices, and improved access to dental care among individuals in higher socioeconomic brackets [9,10]. The role of socioeconomic determinants as independent factors remains ambiguous, as existing evidence indicates that the availability of manpower and access to dental services do not consistently align with improved health outcomes [11].

Findings from the National Pathfinder Survey indicate a pronounced prevalence of periodontal diseases in Nepal, with age, lifestyle habits, and socioeconomic conditions being pivotal factors [12,13]. Limited prior research focusing on the Nepalese population has investigated the impact of SES determinants and variations in lifestyle factors on trends in oral hygiene and periodontal disease among adults. The aim of this cross-sectional study was to assess the association between socioeconomic determinants (income, education, and occupation), oral hygiene practices, and lifestyle factors (smoking, smokeless tobacco, alcohol use, body mass index (BMI), and diabetes) with periodontal disease indicators, namely, bleeding on probing (BOP) and probing depth (PD), in Nepalese adults.

## Materials and methods

Study design and characteristics

An analytical, cross-sectional study was conducted using an interview-administered questionnaire to collect socio-demographic and lifestyle details, followed by periodontal clinical examination.

Study setting and study population

The target convenience sample population was drawn from the Outpatient Department of Periodontics, Institute of Medicine, Tribhuvan University, in Kathmandu, Nepal. Participants aged between 20 and 74 years with no known systemic disease (apart from diabetes mellitus) and those who agreed to participate in the study and sign a written informed consent were included in the study. Participants with aggressive periodontitis or with a previous history of or currently on radiotherapy and/or chemotherapy, pregnant or lactating mothers, patients taking antimicrobials or long-term anti-inflammatory drugs in the previous six months, or patients who underwent periodontal therapy within the last three months were excluded. 

Ethical consideration

This study was performed per the principles stated in the Declaration of Helsinki. It was approved by the Institutional Review Committee of the Institute of Medicine, Tribhuvan University (approval number: 192(6-11) E2 078/079). The study's objectives were thoroughly explained to all participants, who then signed a written informed consent form in English and Nepali, indicating their full understanding and agreement to participate in the study. Participants were allowed to refuse participation or withdraw from the study at any time without any consequences.

Data collection

A total of 374 participants were recruited in this study. Data on demographics included age (categorized as 20-34, 35-44, and 45-65 years) and gender. Oral hygiene status, socioeconomic determinants (income, education, and occupation), and lifestyle factors (BMI, smoking, alcohol consumption, and diabetes mellitus) were recorded using a structured questionnaire and interview, which was filled out for each patient by the examiner. SES was assessed and categorized into upper class (26-29), middle class (11-25), and lower class (1-10) [14]. The level of education was divided into "low" (illiterate, primary, middle, and high school) and "high" (post-high school diploma, graduates and post-graduates, and professionals). Household income was categorized into two groups: >48751 Nepalese rupees (NPR) and <48750 NPR. Occupation was grouped into two levels: I (professionals and semi-professionals) and II (unskilled workers, shop owners, and farmers).

BMI was calculated as body weight (kg) divided by height (m^2^). It was classified into two different groups: normal weight and obese/overweight. To ensure objectivity, participants were asked direct questions regarding their use of tobacco and alcohol consumption. Smoking was categorized as never and current smokers. Current smokers were defined as subjects who smoked more than five cigarettes per day for the past two years or more [15]. Subjects consuming smokeless tobacco were assessed as never smokers and consuming smokeless tobacco daily. Alcohol consumption was assessed as the frequency of consumption of alcoholic drinks and grouped as never and ≥2 times/week. Diabetes mellitus status was assessed as the presence or absence of the disease.

Oral hygiene behaviors included the frequency of daily tooth brushing. This was followed by a periodontal clinical examination that lasted 25-30 minutes on average and included measurement of periodontal disease indicators as BOP and PD. The oral hygiene status was determined via the Simplified Oral Hygiene Index (OHI-S) developed by Greene and Vermillion, consisting of two components of debris and calculus, each with a possible score range of 0-3 [16].

Bleeding is one of the most reliable parameters in evaluating periodontal status [17]. BOP was inspected for four sites of every tooth (distobuccal, mesiobuccal, mesiolingual, distolingual), and the presence or absence of bleeding was noted in an evaluation chart. The absence of BOP served as a predictor of periodontal stability. According to the new classification of periodontal diseases [18], the percentage of sites with >10% bleeding provided information on the presence of gingivitis. PD was categorized as follows: maximum PD ≤4 mm as stage I (mild), maximum PD ≤5 mm as stage II (moderate), and maximum PD ≥6 mm as stage III (severe). The University of North Carolina 15 mm (UNC-15) Color-Coded Probe (Hu-Friedy, Chicago, Illinois, United States) with a black band for each millimeter up to 15 millimeters was utilized to investigate six sites of every tooth, including mid-buccal, distobuccal, mesiobuccal, mesiolingual, distolingual, and mid-lingual, to investigate PD. After periodontal clinical examination, all individuals were provided with the necessary periodontal therapy as per their periodontal treatment plan.

Sample size calculation

The sample size was calculated by using the formula n=z^2^pq/e^2^, where n is the sample size, z is 1.96 at a 95% confidence interval, p is the prevalence of the condition (BOP=71.77%), q is 100-p, and e is the permissible error, which is 5%. Putting these values in the above formula, the sample size calculated was 311.33 participants. Adding 20% of the non-response rate, the total sample size was 373.59 participants [19].

Statistics

Data were entered into a Microsoft Excel Sheet 2010 (Microsoft Corporation, Redmond, Washington, United States) and transferred to IBM SPSS Statistics for Windows Version 24.0 (IBM Corp., Armonk, New York, United States) for statistical analysis. Frequency distribution and percentage were calculated for categorical variables. Bivariate analysis was performed using appropriate tests (chi-squared test/Fisher's exact test) to determine the potential associations between different sociodemographic and lifestyle factors with BOP and PD. The variables that were significantly associated with BOP (yes/no) and the variables with p<0.20 from the bivariate analysis were included in the multivariate binomial logistic regression analysis. Accordingly, the variables that were significantly associated with PD (mild/moderate/severe) and those with p<0.20 from the bivariate analysis were included in the ordinal logistic regression analysis. The level of significance was set at p<0.05.

## Results

Three hundred and seventy-four patients with a mean age of 42.40±12.51 years were included in the study. Two hundred and twenty-two patients (59.4%) were males, while 152 (40.6%) were females (Table 1). 

**Table 1:** Socio-demographic and lifestyle characteristics of the study individuals (n=374) NPR: Nepalese rupees; OHI-S: Simplified Oral Hygiene Index; BMI: body mass index

Variables	Category	Number of participants (%)
Age in years	<35	108 (28.9)
	35-44	97 (25.9)
	45 and above	169 (45.2)
Gender	Male	222 (59.4)
	Female	152 (40.6)
Education	High	123 (32.9)
	Low	251 (67.1)
Income	>48751 NPR	90 (24.1)
	<48750 NPR	284 (75.9)
Occupation	Level I	103 (27.5)
	Level II	271 (72.5)
Socio-economic status	Low	104 (27.8)
	Middle	243 (65)
	Upper	27 (7.2)
Brushing frequency per day	Once	240 (64.2)
	Twice	134 (35.8)
OHI-S	Poor	190 (50.8)
	Fair	144 (38.5)
	Good	40 (10.7)
BMI	Normal weight	253 (67.6)
	Overweight/obese	121 (32.4)
Current smoker	Yes	62 (16.6)
	No	312 (83.4)
Smokeless tobacco consumption	Yes	30 (8)
	No	308(82.4)
Alcohol consumption	>2 times/week	66 (17.6)
	Never	352 (94.1)
Diabetes	Yes	52 (13.9)
	No	322 (86.1)

The occurrence of periodontitis was found to be 62.3%, with 30.7% of individuals having gingivitis. Overall, more than half of the study population had generalized BOP, with 17.6% suffering from severe PD (Table 2).

**Table 2:** Clinical periodontal parameters of the study individuals

Variables	Number of study participants	Category	Number of participants (%)
Periodontal disease status	374	No periodontal disease	26 (7)
		Gingivitis	115 (30.7)
		Periodontitis	233 (62.3)
Bleeding on probing	374	Absent	26 (7)
		Localized	70 (18.7)
		Generalized	277 (74.3)
Probing depth among those with periodontitis	233	Stage I (mild)	77 (33)
		Stage II (moderate)	115 (49.4)
		Stage III (severe)	41 (17.6)

BOP was significantly associated with age, brushing frequency, and OHI-S. However, no association was observed between SES, its determinants with BOP. Among the lifestyle factors, smokeless tobacco showed a significant association (Table 3).

**Table 3:** Association of socio-demographic and lifestyle variables with BOP BOP: bleeding on probing; NPR: Nepalese rupees; OHI-S: Simplified Oral Hygiene Index; BMI: body mass index

Variables	Category	BOP present n (%)	BOP absent n (%)	P-value	Chi-squared value	Test used
Age in years	<35	70 (64.8)	38 (35.2)	0.015	8.642	Chi-squared test
	35-44	71 (73.2)	26 (26.8)			
	45 and above	136 (80.5)	33 (19.5)			
Gender	Male	169 (76.1)	53 (23.9)	0.272	1.209	Chi-squared test
	Female	108 (71.1)	44 (28.9)			
Education	High	94 (76.4)	29 (23.6)	0.466	0.531	Chi-squared test
	Low	183 (72.9)	68 (27.1)			
Income	>48751 NPR	70 (77.8)	20 (22.2)	0.356	0.851	Chi-squared test
	<48750 NPR	207 (72.9)	77 (27.1)			
Occupation	Level I	78 (75.7)	25 (24.3)	0.651	0.205	Chi-squared test
	Level II	199 (73.4)	72 (26.6)			
Socioeconomic status	Lower	79 (76)	25 (24)	0.870	0.279	Chi-squared test
	Middle	178 (73.3)	65 (26.7)			
	Upper	20 (74.1)	7 (25.9)			
Brushing frequency	Once a day	187 (77.9)	53 (22.1)	0.023	5.176	Chi-squared test
	Twice a day	90 (67.2)	44 (32.8)			
OHI-S	Poor	176 (93.6)	12 (6.4)	<0.001	103.299	Chi-squared test
	Fair	91 (63.6)	52 (36.4)			
	Good	10 (23.3)	33 (76.7)			
BMI	Normal weight	181 (73.3)	66 (26.7)	0.629	0.233	Chi-squared test
	Overweight/obese	96 (75.6)	31 (24.4)			
Smoking status	Non-smoker	225 (72.1)	87 (27.9)	0.054	3.721	Chi-squared test
	Current smoker	52 (83.9)	10 (16.1)			
Smokeless tobacco	Never	250 (72.7)	94 (27.3)	0.038	4.312	Chi-squared test
	Current	27 (90)	3 (10)			
Alcohol	Never	233 (72.4)	85 (27.6)	0.113	2.508	Chi-squared test
	>2 times/week	54 (81.8)	12 (18.2)			
Diabetes	Present	42 (80.8)	10 (19.2)	0.234	1.414	Chi-squared test
	Absent	235 (73)	87 (27)			

A multivariate logistic regression model was developed considering BOP, age, brushing frequency, OHI-S, and smokeless tobacco, based on the results of bivariate analysis. This analysis revealed that participants with poor oral hygiene had a markedly increased likelihood of BOP (Table 4).

**Table 4:** Multivariate logistic regression model for bleeding on probing CI: confidence interval; OR: odds ratio; OHI-S: Simplified Oral Hygiene Index

Variables in equation	Categories	β coefficient	P-value	Adjusted OR	95% CI for OR	
					Lower	Upper
Age in years	<35	Reference value				
	35-44	0.106	0.771	1.112	0.543	2.277
	45 and above	-0.104	0.755	0.901	0.467	1.737
Brushing frequency	Once a day	-0.360	0.208	0.698	0.399	1.221
	Twice a day	Reference value				
OHI-S	Good	Reference value				
	Fair	-3.783	<0.001	0.023	0.009	0.058
	Poor	-2.753	<0.001	0.173	0.078	0.386
Smoking	Non-smoker	Reference value				
	Current smoker	0.360	0.406	1.433	0.614	3.346
Smokeless tobacco	Never	Reference value				
	Current consumer	0.348	0.606	1.416	0.378	5.311

The bivariate and multivariable ordinal regression model for PD was limited to 233 individuals. Bivariate analysis revealed age, BOP, and OHI-S, along with lifestyle factors such as smoking and smokeless tobacco, to be statistically significant with severe PD. Among the socioeconomic determinants, unemployed, unskilled workers (17.7%) and less educated (19%) groups had a significant proportion of individuals with stage I, II, and III PD as compared to occupational class I and highly educated individuals. Also, low-income individuals had a high prevalence of deep PD (19.2%) when compared to high-income individuals (1.6%). Overall, severe PD were seen more in lower SES individuals as compared to higher SES individuals (Table 5).

**Table 5:** Association of socio-demographic factors with probing depth NPR: Nepalese rupees; BMI: body mass index; OHI-S: Simplified Oral Hygiene Index

Variables	Category	Periodontitis n (%)			Chi-squared value/Fisher's exact value	P-value	Test used
		Stage I (mild)	Stage II (moderate)	Stage III (severe)			
Age in years	<35	23 (60.5)	11 (28.9)	4 (10.5)	19.428	0.001	Chi-squared test
	35-44	25 (36.2)	34 (49.3)	10 (14.5)			
	45 and above	29 (23)	70 (55.6)	27 (21.4)			
Gender	Male	45 (30.6)	74 (50.3)	28 (19)	1.269	0.530	Chi-squared test
	Female	32 (37.2)	41 (47.7)	13 (15.1)			
Education	High	29 (36.3)	39 (48.8)	12 (15)	0.854	0.652	Chi-squared test
	Low	48 (31.4)	76 (49.7)	29 (19)			
Income	>48751 NPR	23 (34.8)	34 (51.5)	9 (1.6)	0.988	0.607	Chi-squared test
	<48750 NPR	54 (32.3)	81 (48.5)	32 (19.2)			
Occupation	Class I	29 (38.7)	33 (44)	13 (17.3)	1.704	0.427	Chi-squared test
	Class II	48 (30.4)	82 (51.9)	28 (17.7)			
Socioeconomic status	Lower	19 (28.4)	35 (52.2)	13 (19.4)	6.139	0.189	Chi-squared test
	Middle	54 (37.8)	64 (44.8)	25 (17.5)			
	Upper	4 (17.4)	16 (69.6)	3 (13)			
BMI	Normal weight	50 (36.2)	66 (47.8)	22 (15.9)	1.726	0.422	Chi-squared test
	Overweight/obese	27 (28.4)	49 (51.6)	19 (20)			
Brushing frequency	Once a day	52 (32.7)	78 (49.1)	29 (18.2)	0.144	0.930	Chi-squared test
	Twice a day	25 (33.8)	37 (50)	12 (16.2)			
Smoking status	Non-smoker	68 (38)	86 (48)	25 (14)	11.760	0.003	Chi-squared test
	Current smoker	9 (16.7)	29 (53.7)	16 (29.6)			
Alcohol	Never	73 (33.5)	108 (49.5)	37 (17)	1.110	0.675	Fisher's exact test
	>2 times/week	4 (26.7)	7 (46.7)	4 (26.7)			
Smokeless tobacco	Never	75 (35.4)	99 (46.7)	38 (17.9)	7.376	0.025	Chi-squared test
	Current	2 (9.5)	16 (76.2)	3 (14.3)			
Diabetes	Not present	65 (33.7)	94 (48.7)	34 (17.6)	0.233	0.890	Chi-squared test
	Present	12 (30)	21 (52.5)	7 (17.5)			
Bleeding on probing	Not present	20 (50)	17 (42.5)	3 (7.5)	7.457	0.024	Chi-squared test
	Present	57 (29.5)	98 (50.8)	38 (19.7)			
OHI-S	Poor	36 (25.4)	79 (55.6)	27 (19)	13.913	0.004	Chi-squared test
	Fair	38 (45.2)	35 (41.7)	11 (13.1)			
	Good	3 (50)	1 (16.7)	2 (33.3)			

A multivariable ordinal logistic regression model for PD revealed age with an estimate of 1.286 (p=0.001) and BOP with an estimate of 0.811 (p=0.03) to be associated with worse PD. Additionally, lifestyle factors such as current smokers with an estimate of 0.991 (p=0.002) further elevated the risk of severe probing pockets among individuals (Table 6).

**Table 6:** Multivariable ordinal logistic regression model for probing depth CI: confidence interval; OHI-S: Simplified Oral Hygiene Index

Variables in equation	Category	Estimates	Standard error	P-value	95% CI	
					Lower	Upper
Probing depth threshold	Stage I (mild)	0.313	0.908	0.731	-1.467	2.093
	Stage II (moderate)	2.855	0.927	0.002	1.039	4.672
	Stage III (severe)	Reference value				
Age in years	45 and above	1.286	0.394	0.001	0.514	2.059
	35-44	0.972	0.414	0.019	0.161	1.784
	<35	Reference value				
Socioeconomic status	Lower	-0.402	0.485	0.407	-1.353	0.549
	Middle	-0.552	0.449	0.219	-1.431	0.328
	Upper	Reference value				
Smoking status	Current smoker	0.991	0.314	0.002	0.375	1.607
	Non-smoker	Reference value				
Smokeless tobacco	Present	0.341	0.452	0.451	-0.545	1.226
	Absent	Reference value				
Bleeding on probing	Yes	0.811	0.374	0.030	0.078	1.544
	No	Reference value				
OHI-S	Poor	-0.292	0.762	0.702	-1.786	1.202
	Fair	-0.538	0.768	0.483	-2.042	0.966
	Good	Reference value				

## Discussion

This cross-sectional study presents the first data on periodontal parameters concerning SES and its determinants, alongside lifestyle factors in the context of Nepal. The results of this research indicate that nearly 93% of the participants exhibited some form of periodontal disease, with periodontitis being notably prevalent in 62.3% of the individuals. Multivariate analysis identified poor oral hygiene to be significantly associated with BOP. A proportion of participants (17.6%) exhibited severe PD, which were significantly linked to factors such as age, BOP, and tobacco consumption. Conversely, no significant associations were identified with BMI, alcohol intake, smokeless tobacco, and diabetes mellitus. 

The presence of gingival bleeding, along with the progression of PD, should be regarded as indicators warranting specialized periodontal intervention for patients. BOP serves as a widely recognized clinical indicator of periodontal health status and disease progression [20]. Clinical attachment loss is generally regarded as the gold standard for diagnosing and evaluating periodontal conditions. The assessment of gingival bleeding and PD may prove to be an effective methodology in streamlining and simplifying the data collection process for periodontal evaluations, treatment needs, and the implementation of preventive strategies to avert further progression of periodontal disease.

A limited number of studies have examined BOP as a dependent variable. Zimmermann et al.'s work in 2015 [19] emphasized the importance of the associations between SES and its determinants and lifestyle factors, revealing a stronger association with PD as opposed to BOP, thereby aligning with the conclusions drawn in our study. BOP is a significant predictor of periodontal deterioration and is subject to the influences of oral hygiene practices. The literature robustly documents that inadequate oral hygiene is correlated with heightened BOP levels, indicating that the accumulation of bacterial plaque plays a pivotal role in exacerbating gingival bleeding. Alminana-Pastor et al. and Räisänen et al. [21,22] collectively concluded a close association between BOP and poor oral hygiene, which aligns with the findings presented in the current study.

Age is a non-modifiable risk factor associated with periodontal diseases and is extensively discussed in academic literature and various epidemiological studies [23]. In a 2022 study, Carasol et al. identified a significant association between age and periodontal disease, revealing an odds ratio (OR) of 1.43 [24]. The present study reflects this trend with an estimate of 1.28. The cumulative impact of inflammatory cells places older individuals at an elevated risk of periodontium destruction [25]. BOP serves as an indicator of soft tissue inflammation and is also predictive of subsequent periodontal decline. Research has demonstrated that BOP is more prevalent at sites where the periodontal pocket depth is greater than or equal to 6 mm [26]. This observation suggests that deeper pockets are correlated with more severe inflammation and tissue damage. Checchi et al. in 2009 [27] and Zimmermann et al. in 2015 [19] concluded a robust and significant association between BOP and deeper PD, with our findings aligning with their research conclusions.

On analyzing SES, existing evidence on the association between SES and its determinants (occupational class, level of education, and income) reveals an inverse relationship with the severity of periodontal disease [8,28,29]. It is a common belief that individuals in upper socioeconomic classes have healthier behavior, an available supply of health professionals, dental awareness, and better lifestyles than do people in lower classes [11,30].

The findings of this study revealed comparable proportions of patients exhibiting severe PD across both upper and lower occupational classes as well as educational backgrounds. This phenomenon is likely attributable to the limited number of upper-class patients (those with advanced education and professional occupations) who sought care at this tertiary facility, primarily attending for symptomatic relief [1]. Although this study suggested a pronounced effect of low income on severe PD among families with lower household income in comparison to their higher-income counterparts, the findings, nonetheless, did not achieve statistical significance. The observed discrepancy may stem from the fact that even individuals with limited educational backgrounds and non-professional occupations may have incomes exceeding >50,000 NPR, as these individuals often engage in multiple part-time employment opportunities (including shop ownership and various skilled and unskilled labor positions). A higher income level may indeed serve as a contributing factor to the increased frequency of dental visits. This facet warrants further research inquiry into whether oral hygiene and periodontal conditions have improved within the remittance-receiving group, thereby underscoring the necessity for targeted initiatives aimed at enhancing periodontal health among low-income individuals.

Despite the scarcity of research examining social inequalities in oral health in developing countries, a few national surveys from such countries yield conflicting evidence. Hence, we can debate whether there is an evolving trend in attitudes towards oral health among individuals belonging to higher socioeconomic strata. Nevertheless, after controlling for additional risk factors, including smoking and suboptimal oral hygiene, lower SES, in isolation, does not appear to elevate the risk for periodontitis. This raises the question of whether the presumption of a positive correlation between periodontal disease and socioeconomic class is indeed valid within the context of Nepal.

Tobacco smoking potentially modifies inflammatory responses in periodontal tissues and hence is the primary driver of periodontal disease. It can increase the risk up to five- to 20-fold and is associated with greater levels of bone loss, attachment loss, deep periodontal pockets, and tooth loss. The findings of this study showed smoking to be positively associated with deeper PD, with an estimate of 0.99. Also, most of the studies indicate that the presence of diabetes has an impact on the immune reaction of the periodontium [25]. Additionally, poor glycemic control is linked to increased disease progression. However, this study found no association between diabetes and deeper PD. One of the reasons may be the low number of diabetic patients enrolled in this study, and also, 55% of diabetic individuals had good glycemic control in this study [25]. Also, to obtain reliable inferences about causal factors other than smoking, studies need to be restricted to those who have never smoked.

There are mixed conclusions regarding the correlation between BMI and periodontal health. Evidence suggests that this relationship is complex and can vary based on population characteristics and study design. Our study could not establish this association as a dose-response value was not evaluated in this study.

Nepal ranks among the nations grappling with severe periodontal health issues, necessitating immediate attention to oral health services and education. Therefore, it is imperative to acknowledge the significance of social determinants and lifestyle factors concerning periodontal care. Addressing these risk factors and determinants through strategically targeted dental care initiatives, patient education, or community outreach can foster a more inclusive approach to healthcare.

Several limitations of this study are acknowledged. First is the potential influence of confounding variables on self-reported socio-demographic information. Second is the risk of misclassification due to the exclusion of clinical attachment loss as a parameter for assessing periodontal disease. Lastly, as a cross-sectional study, it is inherently limited in its ability to elucidate causal relationships between variables and disease indicators. Despite these limitations, the study furnishes foundational information that can be leveraged to plan oral health promotion strategies for this population group.

## Conclusions

This study identified a positive link between poor oral hygiene and unfavorable lifestyle factors, such as smoking with BOP and severe PD. The findings suggest certain disparities in socioeconomic factors, such as income, that may impact periodontal health. This suggests that while the presence of deep pockets is widespread, further longitudinal studies are required to elucidate the progression of periodontitis in the context of socioeconomic and lifestyle factors.
